# Expression of inflammatory factors and distribution of pathogens in patients with septic shock and their correlation with prognosis: a cross-sectional study

**DOI:** 10.1590/S1678-9946202567045

**Published:** 2025-07-07

**Authors:** Jialin Chen, Mengzhu Tan, Tao Xiang

**Affiliations:** 1 The Third People’s Hospital of Chengdu, Emergency Department, Chengdu,Sichuan, China; 2 Chongqing Medical University, The First Affiliated Hospital, Nephrology Department, Yuzhong, Chongqing, China

**Keywords:** Emergency department of internal medicine, Septic shock, Inflammatory factors, Pathogens, Prognosis

## Abstract

This study aimed to investigate the relationship between inflammatory factors, pathogen distribution, and prognosis in patients with septic shock admitted to the emergency department of internal medicine. A total of 140 patients with septic shock admitted to the emergency department of The Third People’s Hospital of Chengdu, China, from January 2021 to January 2023 were selected for analysis of the distribution of pathogens and infection sites. Patients were divided into death (36 cases) and survival groups (104 cases) based on their condition after 28 days of treatment. A total of 174 pathogenic bacteria strains were cultured, including 124 Gram-negative bacteria (71.26%) and 43 Gram-positive bacteria (24.71%). The main infection sites were the lungs (33.57%) and the abdominal and gastrointestinal tracts (25.00%). The distribution of pathogens and infection sites in the survival and death groups were compared (P > 0.05). The regression equation predicted patient mortality, with the area under the ROC curve of 0.860 (95% CI: 0.792–0.929; P < 0.05), and sensitivity and specificity of 88.90% and 73.10%, respectively. Pathogen distribution in patients with septic shock in the internal medicine emergency department was not related to prognosis; however, inflammatory factors such as CRP and PCT were predictive of outcomes. Along with age, APACHE II score, and SOFA score, they demonstrated significant prognostic value for patient mortality.

## INTRODUCTION

Septic shock is a severe manifestation of systemic inflammatory response syndrome or sepsis, characterized by shock caused by infection. Pathogenesis involves the translocation of pathogenic microorganisms and their metabolic products, such as toxins, from a localized infection site into the bloodstream. This triggers a robust inflammatory response, which subsequently affect various systems and organs, leading to pathological changes^1-3^. Clinical manifestations include hypotension, respiratory distress, and altered consciousness. In severe cases, it can lead to multiple organ dysfunction syndrome and become life-threatening events^4-6^. In recent years, advances in medical technology have significantly reduced the mortality rate of septic shock. However, a substantial number of patients still experience poor prognosis^7-9^.

C-reactive protein (CRP) is a pentameric protein synthesized by the liver, whose levels rise in response to inflammation. As an acute-phase reactant, CRP is primarily induced by interleukin-6 (IL-6), which activates the transcription of the CRP gene during the acute phase of an inflammatory/infectious process^10,11^. Procalcitonin (PCT) level increases in cases of severe bacterial infections, sepsis, and multiple organ dysfunction syndrome, and is related to the immune response of the body^12,13^. Studies suggest that the risk of septic shock varies depending on the type of infection, with Gram-negative bacteria being the main pathogens in patients with septic shock^14,15^. This study investigates the relationship between inflammatory factors, pathogen distribution, and patient prognosis in cases of septic shock treated in the internal medicine emergency department.

## MATERIALS AND METHODS

### Study design and participants

This was a cross-sectional study. A total of 140 patients with septic shock admitted to the internal medicine emergency department of our hospital from January 2021 to January 2023 were included in this study. Inclusion criteria: (1) Diagnosis met the criteria outlined in the Chinese Guidelines for Management of Severe Sepsis/Septic Shock: How to see early goal-directed therapy^16^; (2) Confirmed infection by microbial culture; (3) Age>18 years; (4) Informed consent provided by patients or legal guardians. Exclusion criteria: (1) Presence of malignant tumors or autoimmune diseases; (2) Coinfection with tuberculosis, HIV, or hepatitis viruses (including hepatitis A, B, C, D, and E); (3) Use of immunosuppressive or hormone drugs within one week before admission; (4) Time from symptom onset to admission exceeding 24 h.

Power analysis conducted using G*Power software indicated that a minimum of 131 participants would be needed to detect an effect size of 0.80 (power=80%, p=0.05, odds ratio=1.75). Finally, 140 patients were included in the study. Based on their status 28 days after treatment, patients were divided into death (n=36) and survival group (n=104).

### Data collection

Upon admission, 8 ml of peripheral venous blood was collected from all patients and distributed into three vacuum-sealed collection tubes. One blood sample was centrifuged using a low-speed centrifuge (Hunan Kyd Instrumental Co., Ltd.) at 3000 r/min for 10 min. The upper layer of the blood samples was collected, and serum CRP levels were measured by enzymatic rate scattering turbidimetry. Quantum dot-based immunofluorescence chromatography was used to measure serum PCT levels, and the enzyme-linked immunosorbent assay was used to measure IL-6 levels. All reagent kits used were manufactured by Shenzhen New Industry Biomedical Engineering Co., Ltd. The Anthos Labtec Instruments 17550 microplate readers from Australia were used in this study. One blood sample was tested for alanine transaminase (ALT), aspartate transaminase (AST), serum creatinine (Scr), and blood urea nitrogen (BUN) using a 7600 automatic biochemical analyzer (Hitachi Co. Ltd., Japan). Blood routine parameters—including white blood cell count (WBC) and hemoglobin (Hb)—were recorded using a BC-3000 hematology analyzer (Shenzhen Mindray Bio-Medical Electronics Co., Ltd.), based on a second blood sample.

### Pathogen culture

Upon admission, samples of sputum, abdominal effusion, site-specific secretions, and blood were collected from all patients and promptly sent for bacterial culture using the BioFlo 320 fully automated microbial culture system (EPPENDORF, Germany). The French bioMérieux VITEK-2 Compact fully automated microbial identification system was used to isolate and identify pathogenic bacteria. Identification procedures were conducted in accordance with the National Clinical Laboratory Operating Procedures. Quality control strains of *Escherichia coli* ATCC25922, *Pseudomonas aeruginosa* ATCC27853, and *Klebsiella pneumoniae* ATCC700603 were purchased from Hangzhou Microbial Reagent Co., Ltd.

### Treatment and follow-up methods

Upon admission, all patients were immediately given nasal cannula or mask oxygen therapy. Non-invasive ventilation was provided to those with respiratory failure. A venous access was established, and vital signs were monitored. Broad-spectrum antibiotics—such as piperacillin-tazobactam, meropenem, or vancomycin—were initiated prior to pathogen identification and drug susceptibility testing, upon which sensitive antibiotics were administered for treatment. Targeted fluid resuscitation was performed using 30 ml/kg of intravenous crystalloid fluids within the first 3 hours. Vasopressors were administered when the mean arterial pressure was less than 65 mmHg. Resuscitation goals included a central venous pressure of 8–12 mmHg, mean arterial pressure of no less than 65 mmHg, urine output greater than 30 mL:/h, central venous blood oxygen saturation of no less than 0.7, and a decrease in blood lactate levels. Once hemodynamic stability was achieved, enteral nutrition support was provided, while parenteral nutrition was given to patients with gastrointestinal dysfunction intolerance. Stress-induced hyperglycemia and ulcers were actively prevented. Patients were closely monitored after treatment until either death or full recovery.

### Statistical analysis

Statistical analyses were performed using SPSS program (version 22.0, SPSS Inc., Chicago, IL, USA). Variables such as age, body mass index, and APACHE II score were expressed as mean and standard deviation, and comparisons between groups was conducted using the t-test. Gender, comorbidities and other data were expressed as n (%), and differences between groups were assessed using the c^2^ test. Prognostic factors were analyzed by logistic regression analysis. The predictive value was analyzed using the receiver operating characteristic (ROC) curve.

### Ethics

This study was approved by the Ethics Committee of The Third People’s Hospital of Chengdu, Chengdu City, Sichuan Province, China (TPHC-2021). Written informed consent was obtained from all participants, who informed that their participation was voluntary and that they had the right to withdraw at any time without negative consequences. Participants were provided with detailed explanations about the confidentiality of their information. All procedures were conducted in accordance with relevant guidelines and regulations.

## RESULTS

Among the 140 cases, a total of 174 pathogenic strains were cultured, including 124 Gram-negative bacteria (71.26%) and 43 Gram-positive bacteria (24.71%) (Table 1). The main infection sites were the lungs and the abdominal and gastrointestinal tracts, accounting for 33.57% and 25.00% of cases, respectively (Table 2).

**Table 1:** Pathogen distribution.

Pathogenic bacteria	Number of plants	Constituent ratio (%)
**Gram-negative bacteria**	**124**	**71.26**
*Escherichia coli*	45	25.86
*Pseudomonas aeruginosa*	33	18.97
*Klebsiella pneumoniae*	22	12.64
*Acinetobacter baumannii*	12	6.90
*Enterococcus cloacae*	8	4.60
Other	4	2.30
**Gram-positive bacteria**	**43**	**24.71**
*Staphylococcus aureus*	19	10.92
*Staphylococcus epidermidis*	9	5.17
*Streptococcus pneumoniae*	7	4.02
*Enterococcus sp.*	5	2.87
Other	3	1.72
**Fungi**	**7**	**4.02**
*Candida albicans*	4	2.30
*Aspergillus*	2	1.15
*Candida sp.*	1	0.57
Total	174	100.00

**Table 2:** Infection site distribution.

Infection site	Number of cases	Constituent ratio (%)
Lungs	47	33.57
Abdominal and gastrointestinal tracts	35	25.00
Urinary system	24	17.14
Bloodstream	20	14.29
Multiple	14	10.00
Total	140	100.00

No significant differences were observed in the distribution of pathogens and infection sites between the survival and death groups (P > 0.05) (Table 3). However, age, APACHE II score, SOFA score, CRP, PCT, IL-6, and WBC levels were significantly higher in the death group compared to the survival group (P < 0.05). Gender distribution between the groups showed no statistically significant difference (χ^2^ = 0.063, P = 0.801). In the death group, 61.11% of patients were male and 38.89% were female, while in the survival group, 63.46% were male and 36.54% were female (Table 4). Logistic regression analysis was performed using age, APACHE II score, SOFA score, CRP, PCT, IL-6, and WBC as independent variables, with mortality status as the dependent variable. The analysis identified age, APACHE II score, SOFA score, CRP, and PCT as significant predictors of patient mortality (P < 0.05) (Table 5).

**Table 3:** Comparison of pathogenic bacteria distribution and infection sites between the survival and death groups.

Index	Death group (36 cases and 52 strains)	Survival group (104 cases and 122 strains)	χ^2^	*P*
Pathogenic bacteria distribution (strains)				
Gram-negative bacteria	34 (65.38)	90 (73.77)	1.435	0.488
Isolation of positive bacteria	15 (28.85)	28 (22.95)		
Fungus	3 (5.77)	4 (3.28)		
Infection site (cases)				
Lungs	12 (33.33)	35 (33.65)	0.627	0.960
Abdominal and gastrointestinal tracts	8 (22.22)	27 (25.96)		
Urinary system	7 (19.44)	17 (16.35)		
Bloodstream	6 (16.67)	14 (13.46)		
Multiple	3 (8.33)	11 (10.58)		

**Table 4:** Comparison of clinical data and inflammatory factors between the survival and death groups.

Data	Death group (36 cases)	Survival group (104 cases)	t/χ^2^	*P*
Gender				
Male	22 (61.11)	66 (63.46)	0.063	0.801
Female	14 (38.89)	38 (36.54)		
Age (years)	62.14±5.43	58.89±4.97	3.302	0.001
Body mass index (kg/m^2^)	22.14±2.64	22.03±2.70	0.212	0.833
Hypertension	20 (55.56)	51 (49.04)	0.454	0.500
Diabetes	11 (30.56)	34 (32.69)	0.056	0.813
APACHE II score (points)	26.64±4.54	19.15±2.28	12.835	0.000
SOFA score (points)	12.50±2.12	9.60±2.24	6.785	0.000
CRP (mg/L)	156.64±20.14	131.18±19.65	6.658	0.000
PCT (ng/mL)	2.10±0.94	1.45±0.64	4.618	0.000
IL-6 (pg/mL)	20.45±4.65	18.41±3.12	2.955	0.004
ALT (U/L)	90.41±21.54	87.64±20.06	0.701	0.485
AST (U/L)	84.06±19.70	83.32±21.14	0.184	0.854
Scr (mol/L)	134.45±22.24	130.98±21.18	0.836	0.404
BUN (mmol/L)	10.45±2.17	9.87±2.26	1.340	0.182
WBC (×10^9^/L)	21.14±5.64	18.04±4.94	3.127	0.002
Hb (g/L)	116.64±11.08	118.97±10.12	-1.162	0.247

**Table 5:** Logistic regression model analysis.

Factor	β	SE	Walds	*P*	OR (95% *CI*)
Age	0.822	0.211	15.177	<0.0001	2.275 (1.504∼3.440)
APACHE II-score	1.106	0.275	16.175	<0.0001	3.022 (1.763∼5.181)
SOFA score	1.214	0.324	14.039	<0.0001	3.367 (1.784∼6.354)
CRP	0.632	0.187	11.422	<0.0001	1.881 (1.304∼2.714)
PCT	0.544	0.216	6.343	<0.0001	1.723 (1.128∼2.631)
IL-6	0.324	0.445	0.530	0.432	1.383 (0.578∼3.308)
WBC	0.228	0.615	0.137	0.844	1.256 (0.376∼4.193)
Constant term	-46.541	11.546	16.248	<0.0001	–

The logistic regression model, expressed as ln⁡[P/(1−P)]=0.822× age +1.106× APACHE II score +1.214× SOFA score +0.632× CRP +0.544× PCT −46.541 , was used to predict mortality using ROC curve analysis. The results demonstrated an area under the ROC curve of 0.860 (95% CI: 0.792–0.929), P < 0.05. Sensitivity and specificity were 88.90% and 73.10%, respectively (Table 5).

## DISCUSSION

Septic shock, also known as sepsis, is a common critical illness in clinical practice. Pathogens, toxins, and cell wall products from the infectious site enter the bloodstream, activating the cellular and humoral immune systems, leading to the release of numerous cytokines and endogenous mediators. These factors act on organs and systems, causing tissue ischemia, hypoxia, metabolic disorders, and ultimately multiple organ dysfunction, demonstrating an extremely high mortality rate^17-19^. Worldwide, sepsis affects an estimated 48.9 million people annually and accounts for approximately 20% of all deaths^20^. Mortality rates remain high, with 20–30% of patients dying during the acute phase, underscoring sepsis as one of the leading causes of death worldwide^21^. However, even survivors of the initial phase face a persistently elevated risk of mortality after hospital discharge, with about 30% dying within the first year^22^. Current guidelines recommend that patients with septic shock be treated in intensive care units, receiving early antibiotic therapy and fluid resuscitation^23^. Despite these measures, recent challenges such as worsening environmental conditions, antibiotic overuse, and increased bacterial resistance have complicated septic shock treatment^24-26^.

In this study, samples including sputum, abdominal fluid, infection site secretions, and blood were collected from all participants and cultured. A total of 174 strains of pathogens were isolated, of which 124 (71.26%) were Gram-negative bacteria, followed by 43 (24.71%) strains of Gram-positive bacteria. The main infection sites were the lungs and the abdominal and gastrointestinal tracts, accounting for 33.57% and 25.00%, respectively. No statistically significant difference was observed in pathogen distribution and infection sites between the survival and death groups. This suggests that Gram-negative bacteria are more commonly associated with septic shock, with primary infection sites in the lungs and abdominal and gastrointestinal tracts. The type of pathogen and the infection site are not related to patient prognosis, which aligns with previous research^27^.

The APACHE II and SOFA scores are commonly used tools to evaluate the condition and prognosis of critically ill patients in clinical practice^28-30^.

Numerous inflammation-related biomarkers have been documented, including WBC, neutrophils percentage (NE%), CRP, erythrocyte sedimentation rate (ESR), IL-6 and IL-10, PCT, platelet count (PLT), vascular endothelial growth factor (VEGF), tumor necrosis factor-α (TNF-α), macrophage inflammatory protein-1β (MIP-1β), IL-2, and IL-8^31,32^. Among these markers, WBC, CRP, NE%, PLT, ESR, PCT, IL-6, and IL-10 have been identified as potential diagnostic biomarkers for bloodstream infections^32^.

This study found that age, APACHE II score, SOFA score, and the levels of CRP, PCT, IL-6, and WBC were significantly higher in the death group compared to the survival group. This is due to the fact that older patients experience degenerative changes across various systems and organs and exhibit diminished functional compensation after septic shock, making them more prone to organ failure and death. Higher APACHE II and SOFA scores reflect more severe conditions and are associated with poorer prognosis. CRP, an acute-phase reactant protein synthesized by the liver, can activate complement and mononuclear macrophages, clearing pathogens or damaged cells. CRP levels typically begin to rise within approximately 6 hours after the onset of infection or inflammation^33^. PCT can be secreted by all parenchymal tissue cells in response to infection, causing rapid increase in its blood concentration^34^. IL-6 is key cytokine network, which can mediate acute-phase reaction of the liver and promote secretion of CRP and PCT^35^. WBC is an important cell of the body’s immune system, which increases during infection and inflammation and can eliminate pathogens by phagocytosis and antibody production^36,37^. Higher levels of CRP, PCT, IL-6, and WBC indicate more severe inflammatory response, which may result in more extensive organ damage and poorer prognosis.

Gender distribution analysis between groups revealed no statistically significant difference. These findings suggest that gender alone may not be determinant on mortality outcomes in the studied population. However, this observation does not negate the potential influence of gender-related biological and physiological factors on the progression and management of septic shock.

Gender differences in immune responses and inflammatory regulation are well-documented, with males and females having distinct hormonal influences that could affect cytokine release, immune cell activity, and systemic inflammation. For instance, estrogen in females has been associated with anti-inflammatory effects, while testosterone in males may modulate immune responses differently^38^.

In this study, age, APACHE II score, SOFA score, CRP, PCT, IL-6, and WBC were used as independent variables, and death was used as the dependent variable for logistic regression analysis. The results showed that age, APACHE II score, SOFA score, CRP, and PCT were all influencing factors affecting patient mortality. The regression equation predicted patient mortality with a ROC curve area of 0.860, 88.90% sensitivity, and 73.10% specificity. This suggests that older patients with higher APACHE II score, SOFA score, CRP, and PCT levels are at a higher risk of mortality from septic shock. Such patients should be regarded as high-risk groups in clinical practice, and their vital signs should be closely monitored with effective emergency measures to reduce mortality risk.

### Strengths and limitations

This study has several strengths, including comprehensive data collection, the use of standardized tools like APACHE II and SOFA scores, and a robust statistical approach to identifying prognostic factors such as age, CRP, and PCT. The findings provide clinically relevant insights into risk stratification and pathogen distribution in patients with septic shock, broadening the understanding of this critical condition. However, the study is subject to several limitations that should be considered.

Firstly, the study was conducted in a single specialized hospital in Chengdu, which may limit the results generalization to other healthcare settings or regions with different populations and clinical practices. Although 140 patients composed the sample, it remains relatively small, and no formal power analysis was performed, potentially limiting the ability to detect subtle differences between groups. Additionally, the study was based on the 2014 Chinese Guidelines for the Management of Severe Sepsis and Septic Shock, which slightly differ from the internationally accepted Sepsis-3 definitions. This may affect comparability with studies that adopted the latter.

Moreover, while pathogens and infection sites were documented, no explicit analysis was made to correlate specific microorganisms with their respective infection sites, which may limit the clinical applicability of the findings. Notably, antimicrobial resistance profiles were not analyzed, which could have influenced clinical outcomes and mortality. Furthermore, excluding patients with certain comorbidities narrows the applicability of the results to specific populations. The lack of long-term follow-up and limited assessment of treatment variability leaves critical aspects of patient outcomes unexplored. Potential confounding variables and the absence of multi-center data further limit the external validity of the study.

Lastly, the impact of polymicrobial infections on prognosis was not addressed, despite the possibility that some patients may have had mixed infections. Therefore, conducting multicenter studies with larger sample sizes, resistance profiling, and more detailed analyses of pathogen-site associations is essential to confirm and expand upon these findings. Despite these limitations, the study makes valuable contributions to understanding septic shock prognosis.

## CONCLUSION

In conclusion, pathogen distribution among septic shock patients in the internal medicine emergency department was unrelated to patient prognosis. However, inflammatory biomarkers such as CRP and PCT are influencing factors affecting patient prognosis. When combined with age, APACHE II score, and SOFA score, these biomarkers demonstrated predictive value for mortality. However, examining specific pathogen subsets or multi-microbial infections, along with their interactions with clinical variables, could offer deeper insights into their effects on patient outcomes. Although our study did not focus on these subsets, future research could delve into these relationships, potentially uncovering more precise prognostic implications. Moreover, the significance of pathogen resistance profiles cannot be overstated. Resistance patterns are critical in treatment efficacy and, consequently, prognosis. Future studies incorporating these factors could provide a more comprehensive understanding of the intricate interplay between pathogens, resistance, and clinical outcomes.
